# Diagnostic Value of Whole-Blood and Plasma Samples in Epstein–Barr Virus Infections

**DOI:** 10.3390/diagnostics13030476

**Published:** 2023-01-28

**Authors:** Mateusz Rzepka, Dagmara Depka, Eugenia Gospodarek-Komkowska, Tomasz Bogiel

**Affiliations:** 1 Department of Microbiology, Ludwik Rydygier Collegium Medicum in Bydgoszcz, Nicolaus Copernicus University in Toruń, 85-094 Bydgoszcz, Poland; 2 Department of Clinical Microbiology, Antoni Jurasz University Hospital No. 1, 85-094 Bydgoszcz, Poland

**Keywords:** EBV, EBV DNAemia, Epstein–Barr virus, HHV-4, human herpesvirus 4, molecular diagnostics, plasma, viremia monitoring, whole blood

## Abstract

Epstein–Barr virus (EBV) is an oncogenic virus classified by the World Health Organization as a class 1 carcinogen. Post-transplant lymphoproliferative disorders are believed to be strongly related to an EBV infection. Monitoring of EBV DNAemia is recommended to assess the risk of reactivation of latent infection and to assess the effectiveness of therapy. Currently, various types of clinical specimens are used for this purpose. The aim of the study was to assess a reliable method of EBV viral load investigation depending on the clinical material used: whole blood or plasma samples. We found that of 134 EBV-DNA-positive whole-blood samples derived from 51 patients (mostly hemato-oncology or post-transplantation), only 43 (32.1%) were plasma-positive. Of these, 37 (86.0%) had lower plasma DNAemia compared to the corresponding whole-blood samples. We conclude that whole-blood samples have a higher sensitivity than plasma samples in EBV DNA detection. The clinical utility of the tests is unclear, but our results suggest that either whole blood or plasma should be used consistently for EBV viral load monitoring.

## 1. Introduction

Epstein–Barr virus (EBV), also known as human herpesvirus 4 (HHV-4), belongs to the *Herpesviridae* family, which also includes other prevalent viruses pathogenic to humans, e.g., herpes simplex virus, varicella zoster virus, and cytomegalovirus (CMV). The EBV genome is a double-stranded, linear DNA molecule and encodes nearly one hundred proteins. EBV is one of the most common viruses that infect humans. More than 90% of adults become seropositive for this virus at some stage [1,2,3]. The infection occurs in child- and adulthood, and its manifestation is usually subclinical or mild, such as respiratory infection or fever. Some people (especially adults) develop an acute form of infection—infectious mononucleosis. It is characterized by, among others, pharyngitis, rhinitis, high fever, cervical lymphadenopathy, and hepatosplenomegaly although the occurrence of individual symptoms varies among patients [1,2,4,5]. EBV infection may also cause neurological complications, with the estimated incidence of 5% in children [6,7].

Within the human body, EBV resides mainly in the lymphoid tissue of the throat. Therefore, the ubiquity of the virus in the human population is primarily due to its easy spread, mainly through saliva. This explains the high rate of seroprevalence in adults regardless of geographic region. Other routes of EBV infection transmission include blood transfusion and organ transplantation [1,2,3].

EBV is an oncogenic virus classified by the World Health Organization as a class 1 carcinogen [2]. It is estimated that approximately 200,000 cases of cancer, including gastric carcinoma, nasopharyngeal carcinoma, Hodgkin disease, and Burkitt lymphoma, detected annually are related to EBV infections [5,8,9]. Moreover, lymphomas are among the most common cancers in hematopoietic stem cells and solid-organ recipients [5]. Hematopoietic stem cell transplantation (HSCT) is an important risk factor for post-transplant lymphoproliferative disorder (PTLD). The occurrence of PTLD after HSCT is primarily associated with EBV infection and is characterized with a high mortality rate [10,11,12,13]. Risk factors for EBV-PTLD after HSCT include, among others, T-cell depletion, EBV serology donor/recipient mismatch, cord blood transplantation, human leukocyte antigens mismatch, and splenectomy [10,14].

The main targets of EBV are naive B lymphocytes that differentiate into memory B cells (MBCs), which most commonly results in a persistent infection [2,5,15]. In immune-competent individuals, a proliferation of B cells is controlled by cytotoxic T cells (CD8+), with healthy people having up to 14% of those cells that are specific for various EBV epitopes [16]. However, during immunosuppression, MBCs may become malignant after transforming into a lymphoblastic lineage. Transplant recipients, people with congenital immunodeficiency, or patients infected with human immunodeficiency virus are at risk of reactivation of EBV infection [2,5].

Serological, histopathological, and molecular tests are used for laboratory diagnosis of EBV infection [10,17]. The latter are important since they enable quantitative measurement of EBV DNAemia. Molecular biology methods are recommended to monitor the EBV load in people at risk of latent infection reactivation, especially in patients after HSCT [10,18,19]. In solid-organ transplantation (SOT), EBV DNAemia should be monitored mainly in seronegative recipients in case of a seropositive donor [17,18] in order to prevent and treat PTLD [10,18]. Cell-free EBV DNA level measurement in the blood may also be useful in monitoring treatment in nasopharyngeal cancer or Hodgkin lymphoma [20,21].

Whole blood, plasma, and serum fractions are used to quantify EBV DNAemia [10]. In this study, we use the commercial GeneProof Epstein–Barr Virus (EBV) PCR Kit (GeneProof, Brno, Czech Republic) to compare EBV DNA detection in whole-blood and plasma samples.

## 2. Materials and Methods

### 2.1. Clinical Samples

In the study, 134 plasma samples derived from 51 patients with positive EBV DNA whole-blood samples were analyzed. They included 22 (43.1%) patients after a kidney or liver transplant, 21 (41.2%) after HSCT, 4 (7.8%) with reactivated or suspected infectious mononucleosis, and 1 patient each with either acute respiratory distress syndrome, pericarditis, or cardiac disorders with acute myeloid leukemia and 1 patient with malignant melanoma and hepatitis. The detailed characteristics of patients are presented in Supplementary Materials, Table S1.

The whole-blood samples were collected into BD Vacutainer^®^ K_3_EDTA tubes (Becton, Dickinson and Company, Sparks, MD, USA) from patients hospitalized at Antoni Jurasz University Hospital No. 1 in Bydgoszcz, Poland (Table 1). Repeated specimen collection and testing of whole-blood/plasma samples were performed for 27 (52.9%) patients, with several days between (Table S1). The criterion for the inclusion of the patient in the study was obtaining a positive result for EBV DNA in the former testing. The investigation was performed as part of the routine clinical diagnostics. 

This study was approved by the Bioethics Committee of the Nicolaus Copernicus University in Toruń Collegium Medicum in Bydgoszcz, Poland (Approval Code: KB 239/2021, granted on 23 March 2021).

### 2.2. DNA Extraction

DNA was extracted from all of the whole-blood samples as well as from the corresponding plasma samples.

The first stage of this study involved manual isolation of DNA from whole-blood samples using spin columns with a silica membrane. For this purpose, the commercial GeneProof PathogenFree DNA Isolation Kit (GeneProof, Brno, Czech Republic) was used, and the manufacturer’s instructions were followed. The whole-blood samples were stored up to one day at 4 °C from the moment of delivery to the laboratory.

In the second stage of this study, the whole-blood positive samples used in the first stage were centrifuged (3000 rpm/10 min) to separate plasma fraction. The plasma samples were kept in sterile, DNase- and RNase-free tubes (Eppendorf, Hamburg, Germany), and DNA was isolated as described in the previous step. Plasma fraction was separated within two hours of DNA extraction from the whole-blood samples and frozen at −80 °C. DNA was extracted from the plasma samples within a week of freezing. The plasma specimens were thawed at room temperature (22–25 °C) for 30 min before starting the diagnostic procedure.

In each stage, 100 µL of DNA eluate was obtained, which was then used for nucleic acids amplification.

### 2.3. Nucleic Acid Amplification Test

Immediately after DNA extraction, real-time polymerase chain reaction (PCR) was performed using the GeneProof Epstein–Barr Virus (EBV) PCR Kit—ISEX Version (GeneProof, Brno, Czech Republic) and the Cobas z 480 instrument (Roche, Basel, Switzerland). The real-time PCR temperature profile was applied according to the manufacturer’s instruction (Supplementary Materials, Table S2).

These reactions were performed independently for DNA samples extracted from both whole-blood and plasma specimens. The target sequence was Epstein–Barr virus nuclear antigen 1 (EBNA1), and the detection limit of the assay with the applied DNA extraction method was 196.088 IU/mL (regardless of sample type).

### 2.4. Data Interpretation and Statistical Analysis

The results were interpreted quantitatively and expressed as International Units per milliliter (IU/mL) [22] in relation to the determined calibration curve based on the four standards included in the kit (10^1^, 10^2^, 10^3^, and 10^4^ copies/µL).

A specific shape of the amplification curve for the target sequence (FAM detection channel) and for the internal standard—IS (HEX detection channel)—was classified as a positive result (Supplementary Materials, Figure S1). Some highly loaded EBV DNA samples (>10,000 IU/mL) were classified as positive regardless of the amplification curve for IS, in accordance with the criteria determined by the test manufacturer. A lack of amplification curve in the FAM channel and fluorescence curve in the HEX-detecting channel (threshold cycle <38) was interpreted as a negative result. Positive results below the linearity range (10^2.5^ IU/mL) were interpreted as <320 IU/mL.

Statistical analysis was performed in the Statistica™ 13.3 (TIBCO Software Inc., Palo Alto, CA, USA) program. The Wilcoxon test was used to determine statistically significant differences between the values of EBV DNAemia in whole blood and plasma samples (α = 0.05), while the chi-square test was used to determine the relationship between the result obtained from plasma samples and the sex/age of the patients (α = 0.05). The relationship between positive results from whole=blood and plasma samples was determined using the Pearson’s correlation (α = 0.05).

## 3. Results

EBV DNA was found in all 134 (100%) whole-blood samples chosen according to a selection criteria for the study purpose. Variable viral loads ranging from <320 to 5.5 × 10^6^ IU/mL were detected for these samples. Only 43 (32.1%) samples were positive for EBV DNA when plasma was used for the testing (Table 2).

**Figure 1:**
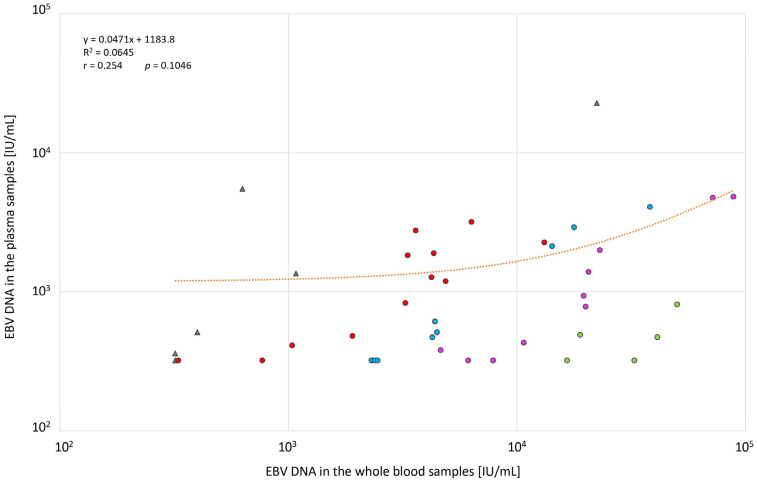
EBV DNAemia (IU/mL) distribution in the whole-blood (*n* = 42) versus the corresponding plasma samples (*n* = 42). The chart does not include the outlier (x = 5,500,000; y = 33,250). Samples representing higher EBV DNAemia values compared to the corresponding plasma samples are marked with red (up to 5 times higher), blue (6–10 times higher), pink (11–25 times higher), and green (>25 times higher) dots. Samples with higher plasma EBV DNAemia values or equal values are marked with gray triangles. The orange line represents the regression curve. *p*, probability value; r, Pearson’s correlation coefficient; R^2^, coefficient of determination.

Among the positive samples in both types of clinical specimens (*n* = 43), 37 (86.0%) had higher values in the whole blood samples, but there was no significant correlation between the two types of samples (Figure 1). The viral load in the whole-blood samples was higher as follows:

- Up to 5 times; 12 (32.4%) samples;
- 6–10 times; 9 (24.3%) samples;
- 11–25 times; 10 (27.0%) samples;
- >25 times; 6 (16.2%) samples.

EBV DNA viral load values detected in five (11.6%) plasma samples were higher than in the corresponding whole-blood samples. However, the values were nearly identical in four (9.3%) samples, with only one presenting a nearly 10-fold higher value in the plasma (Table 3). The detailed results are shown in Supplementary Materials, Table S3.

Overall, the differences in DNAemia values for both types of specimens were one log for 21 (48.8%) samples and two logs for 9 (20.9%) samples. For the remaining 13 (30.2%) samples, no significant differences were noted.

**Figure 2:**
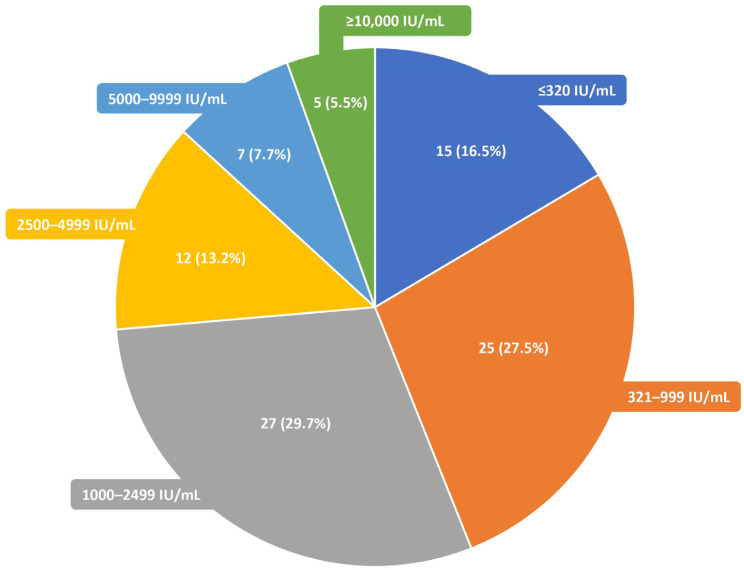
The distribution of EBV DNA load (IU/mL) in the whole blood (*n* = 91) for the corresponding negative plasma samples (*n* = 91).

As many as 91 (67.9%) plasma samples were negative for EBV DNA (Table 2). Of these samples, 40 (44.0%) presented low EBV DNAemia values (<1000 IU/mL) in the corresponding whole-blood samples. However, five (5.5%) of the investigated negative plasma samples showed high EBV DNAemia levels (≥10,000 IU/mL) when the whole-blood samples were used (Figure 2).

No statistically significant differences were noted between the results (either positive or negative) obtained for plasma samples and patients’ sex (χ² = 0.02, *p* = 0.8744) or age (χ² = 2.89, *p* = 0.0892).

## 4. Discussion

Currently, molecular biology methods are commonly used in the diagnostics of EBV infection [23,24,25]. First of all, they enable the quantitative measurement of EBV DNA in the tested clinical specimens, which allows for a detection of the reactivation of latent infection and monitoring the effectiveness of a treatment. This is of great importance for immunosuppressed patients, particularly those after HSCT and SOT [24,26]. However, the threshold point of EBV DNAemia for the initiation of treatment has not yet been established. Instead, changes within ±0.5 or 1 log_10_ were found to be clinically significant when using the same sample types [10,17].

The main finding of this study is that whole-blood samples have a higher sensitivity than plasma samples in detecting EBV DNA using the GeneProof Epstein–Barr Virus (EBV) PCR Kit. EBV DNA was not detected in 67.9% of plasma with positive whole-blood samples. However, most of these specimens had relatively low whole-blood EBV DNA values, which might be due to the presence of a persistent infection, which is not clinically relevant. There were, however, several whole-blood samples with a high EBV DNA load (≥10,000 IU/mL), which is unlikely to be a consequence of a persistent infection and may have clinical utility.

Others have also reported a low correlation between EBV DNA in plasma and whole-blood samples in kidney transplant recipients [27] and in patients after HSCT [28] due to negative results in plasma samples. Thus, follow-up EBV DNA tests need to rely on one type of sample. Several studies have shown similar results but compared diagnostic kits different from those used in this study [24,26,29,30,31,32]. We have also shown previously that whole blood has a higher sensitivity than plasma in detecting CMV DNAemia [33]. 

A strength of this study is that technician variability was minimized by the fact that only one technician handled the samples, including DNA extraction and the real-time PCR, and that only one diagnostic assay was used with the same detection threshold for both types of samples. The limitations of this study were that patients with various disease states were included, and patients’ current treatment and clinical status was not available. 

Finally, the increased sensitivity does not necessarily translate to increased clinical utility. Thus far, no recommendation has been established regarding the type of sample for EBV-DNA-level evaluation [10]. Due to the wide range of diseases associated with EBV, the detection of EBV DNA in plasma or whole blood cannot be a specific diagnostic marker, but it may be a reliable prognostic factor [34,35,36]. Ha et al. [37] reported that the presence of EBV DNA in plasma is significantly associated with advanced extranodal natural killer T-cell lymphoma (ENKTCL), while it is not found for whole-blood samples. Yan et al. [38] showed that the presence of EBV DNA in plasma identified patients at risk for ENKTCL and recurrent disease. On the other hand, no such relationship was demonstrated when peripheral blood mononuclear cells were used for testing [38]. In contrast to plasma samples, detection of EBV DNA in whole blood may be more useful in predicting the response and adverse events of SMILE (steroid, methotrexate, ifosfamide, L-asparaginase, etoposide) therapy in ENKTCL nasal type [39]. Other studies have reported that EBV DNA in whole-blood samples may be a useful prognostic marker in patients with Hodgkin disease [40] or diffuse large B-cell lymphoma [41]. Future studies should focus on trying to establish the relationship between the obtained EBV DNA values from different types of samples in patients with different disorders before, during, and after treatment.

Of note, when interpreting the results of molecular testing, it should be remembered that the EBV DNA in plasma or whole-blood samples is sometimes detected during respiratory tract infections, autoimmune disorders, or others, which may additionally affect the final results of a viral load investigation [42].

## 5. Conclusions

Quantitative measurement of EBV DNA using real-time PCR is an important diagnostic tool for post-transplantation patients. We conclude that whole-blood samples have a higher sensitivity than plasma samples in EBV DNA detection. The clinical utility of the tests is unclear, but our results suggest that either whole-blood or plasma samples should be used consistently for EBV viral load monitoring.

## Figures and Tables

**Table 1:** General characteristics of patients (*n* = 51).

Age Group	Sex	*n*	Percentage
Children	Female	12	23.5%
	Male	12	23.5%
Adults	Female	12	23.5%
	Male	15	29.4%

**Table 2:** Number of EBV DNA detection results in the plasma (*n* = 134) versus the corresponding whole-blood (*n* = 134) samples.

	Whole Blood	Plasma	
	Positive EBV DNA	Positive EBV DNA	Negative EBV DNA
*n*	134	43	91
Percentage	100%	32.1%	67.9%

**Table 3:** The list of EBV DNA levels (IU/mL) for the samples with higher plasma (*n* = 5) DNAemia when compared to the whole-blood counterparts (*n* = 5).

Sample No.	EBV DNA (IU/mL) Level		Difference
	In Whole Blood	In Plasma	
15,729	1080	1350	270
15,679	22,400	22,800	400
18,216	320	360	40
17,343	630	5500	4870
15,407	400	510	110

## Data Availability

The data presented in this study are available on a reasonable request from the corresponding author.

## Supplementary Materials

The following supporting information can be downloaded at: https://www.mdpi.com/article/10.3390/diagnostics13030476/s1, Figure S1: An example of amplification curves for (a) positive (sample number 1) and negative (sample number 2) results (FAM detection channel) and (b) internal standard (HEX detection channel); Table S1: The number of samples taken for testing with patient characteristics; Table S2: An amplification profile applied in the present study (GeneProof Epstein–Barr Virus (EBV) PCR Kit); Table S3: The detailed levels of EBV DNA in the whole blood (*n* = 134) and the corresponding plasma samples (*n* = 134) (IU/mL).
