# Supplementary material for: Diagnostic Value of Whole-Blood and Plasma Samples in Epstein–Barr Virus Infections
Source: Diagnostics (Basel). 2023 Jan 28;13(3):476. doi: 10.3390/diagnostics13030476 (PMC9914079; doi:10.3390/diagnostics13030476)
Supplement: Supplementary file 1 [file diagnostics-13-00476-s001.zip › diagnostics-2147377-supplementary.pdf]

Diagnostic Value of Whole Blood and Plasma Samples in Epstein-Barr Virus Infections

Mateusz Rzepka [1,2,*], Dagmara Depka [1,2], Eugenia Gospodarek-Komkowska [1,2] and Tomasz Bogiel [1,2,*]

[1] Department of Microbiology, Ludwik Rydygier Collegium Medicum in Bydgoszcz, Nicolaus Copernicus University in Toruń, 85-094 Bydgoszcz, Poland
[2] Department of Clinical Microbiology of Antoni Jurasz University Hospital No. 1, 85-094 Bydgoszcz, Poland
* Correspondence: mateusz.rzepka@cm.umk.pl (M.R.), t.bogiel@cm.umk.pl (T.B.), Tel.: +48 52 585 44 80

Table S1. The number of samples taken for testing with patient characteristics.

Number of Whole Blood Samples	Number of Plasma Samples	Patient No.	Age [years]	Sex	Initial Hospitalisation Reason	Disease Diagnosis and/or Risk Factors for EBV Reactivation
7	7	3.	2	Female	Haemato-oncology	ALL, HSCT
5	5	15.	13	Female		CGD, HSCT
4	4	12.	3	Female		SAA, HSCT
4	4	22.	8	Female		SAA, HSCT
4	4	45.	7	Female		HLH, HSCT
3	3	9.	13	Female		SAA, HSCT
3	3	50.	15	Female		ALL, HSCT
2	2	7.	4	Female		Neuroblastoma, HSCT
2	2	30.	8	Female		HTx, EBV infection reactivation
1	1	1.	4	Female		SAA, HSCT
1	1	14.	13	Female		SAA, HSCT
1	1	47.	11	Female		Suspicion of IM
9	9	43.	2	Male		ALL, HSCT
4	4	23.	10	Male		ALL, HSCT
4	4	25.	2	Male		ALL, HSCT
4	4	38.	11	Male		AML, HSCT
3	3	2.	13	Male		AML, HSCT
3	3	10.	13	Male		ALL, HSCT
2	2	24.	19	Male		AML, HSCT
2	2	26.	7	Male		HTx, EBV infection reactivation
2	2	42.	9	Male		hepatitis, melanoma malignum
2	2	51.	2	Male		SCID, HSCT
1	1	8.	13	Male		EBV-related PTLD, HSCT
1	1	21.	6	Male		ALL, HSCT
1	1	39.	2	Male		ALL, HSCT
19	19	40.	55	Female	Transplantology	LTx
4	4	32.	28	Female		LTx
3	3	35.	31	Female		KTx
1	1	11.	50	Female		KTx
1	1	17.	55	Female		KTx
1	1	28.	73	Female		KTx
1	1	36.	36	Female		KTx
1	1	37.	44	Female		KTx
1	1	41.	69	Female		KTx
1	1	49.	77	Female		KTx

5	5	4.	54	Male		LTx
3	3	13.	61	Male		LTx
2	2	5.	44	Male		KTx
1	1	6.	49	Male		KTx
1	1	18.	59	Male		KTx
1	1	27.	61	Male		KTx
1	1	31.	61	Male		KTx
1	1	33.	61	Male		KTx
1	1	34.	25	Male		KTx
1	1	44.	27	Male		LTx
1	1	46.	66	Male		KTx
1	1	48.	46	Male		KTx
3	3	29.	63	Male	Intensive care	ARDS
1	1	19.	27	Female	Cardiology	pericarditis
1	1	16.	81	Male		AML
2	2	20.	23	Female	Nephrology	Suspicion of IM

ALL—acute lymphoblastic leukemia, AML—acute myeloid leukemia, ARDS—acute respiratory distress syndrome, CGD—chronic granulomatous disease, EBV—Epstein-Barr Virus, HLH—hemophagocytic lymphohistiocytosis, HSCT—hematopoietic stem cell transplantation, HTx—heart transplantation, IM—infectious mononucleosis, KTx—kidney transplantation, LTx—liver transplantation, PTLD—post-transplant lymphoproliferative disorders, SAA—severe aplastic anemia, SCID—severe combined immunodeficiency

Table S2. An amplification profile applied in the present study (GeneProof Epstein-Barr Virus (EBV) PCR Kit).

Step	Temperature	Time	Cycles
Hold	37° C	2 min.	1
Hold	95° C	10 min.	1
PCR	95° C	5 sec.	45
	60° C*	40 sec.	
	72° C	20 sec.	

*—data acquisition at FAM and HEX detection channels, PCR—polymerase chain reaction

Table S3. The detailed levels of EBV DNA in the whole blood (n = 134) and the corresponding plasma samples (n = 134) [IU/mL].

Sample No.	EBV DNA [IU/mL]		Sample No.	EBV DNA [IU/mL]		Sample No.	EBV DNA [IU/mL]	
	in Whole Blood	in Plasma		in Whole Blood	in Plasma		in Whole Blood	in Plasma
37242	410	0	1505	5230	0	15704	13,200	2260
15729	1080	1350	19933	12,070	0	16159	17,800	2910
23880	2390	0	26515	690	0	16599	4650	0
24611	690	0	811	320	0	16964	720	0
14831	3250	830	32375	450	0	17430	4370	0
15279	4380	610	33702	1330	0	18117	6050	0
15679	22,400	22,800	42101	800	0	19087	3250	0
16795	88,670	4820	35674	770	320	19918	4350	0
17310	38,200	4080	16590	610	0	20639	6270	0
17701	4330	1890	17343	630	5500	21576	930	0
18216	320	360	18181	14,130	0	22559	1310	0
7466	450	0	19466	320	0	23663	4470	510
7877	3200	0	8463	4640	380	25203	1530	0
8412	12,870	0	8934	23,070	1990	26105	2440	0
8697	6310	0	29230	16,600	320	27066	1200	0
8944	2460	320	29747	7870	320	28198	320	0
27264	4110	0	30672	14,270	2130	28727	770	0
30792	1170	0	31247	1210	0	39845	410	0
26364	1130	0	42637	320	0	29175	4270	470
7420	4200	0	40754	2040	0	39837	1890	0
6556	10,730	430	22541	850	0	40767	2400	0
20958	5,500,000	33,250	26928	1390	0	23732	320	0
29922	6330	3180	28974	19,600	935	25240	320	0
31243	740	0	29638	32,670	320	28194	2390	0
38939	620	0	33163	18,930	490	29684	1510	0
15886	41,200	471	41171	4230	1270	33709	20,000	780
17038	72,000	4750	40025	56,800	0	35528	2310	320
17730	320	0	22027	50,270	810	34582	8800	0
15926	6130	320	29640	2020	0	6661	320	0
21141	790	0	30118	840	0	40801	2850	0
23162	2380	320	30556	320	0	6474	1130	0
24031	330	320	20569	320	0	20713	1160	0
24431	860	0	16920	320	0	27007	1490	0
28200	1060	0	22422	3330	1830	32292	2550	0
28904	320	0	18265	2500	0	37297	360	0
30791	1040	410	33328	1290	0	22670	1830	0
40756	320	0	33434	1290	0	17289	620	0
16578	4880	1190	27064	320	0	29479	320	0
20870	3610	2760	6499	950	0	37688	790	0
24866	830	0	40998	20,600	1390	39411	320	320
32941	1030	0	41673	16,600	0	42902	2610	0
41751	1910	480	42550	6250	0	40764	1710	0
19736	660	0	39478	9000	0	25715	2710	0
21053	1900	0	33661	1310	0	28055	970	0
25835	790	0	15407	400	510			

DNA—deoxyribonucleic acid, EBV—Epstein-Barr Virus, IU—international unit

Figure S1. An example of amplification curves for (a) positive (sample number 1) and negative (sample number 2) results (FAM detection channel) and (b) internal standard (HEX detection channel).

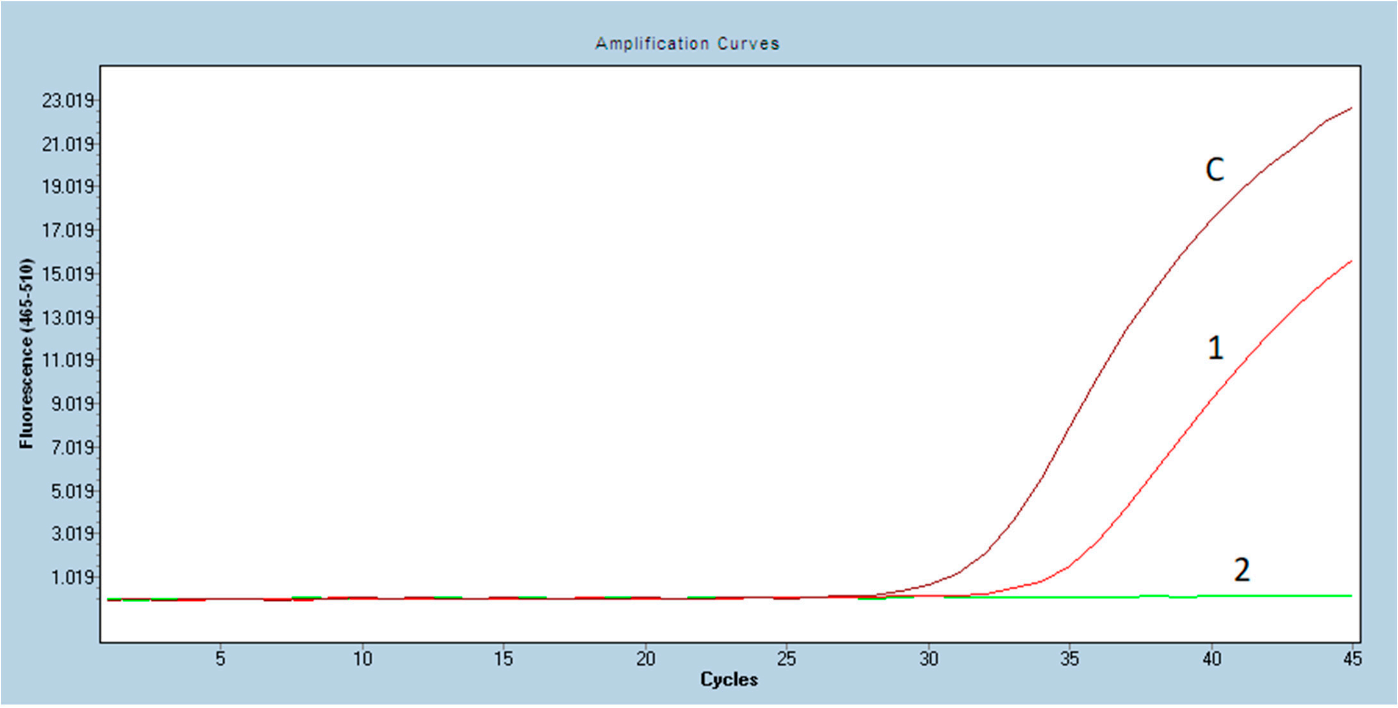

(a)

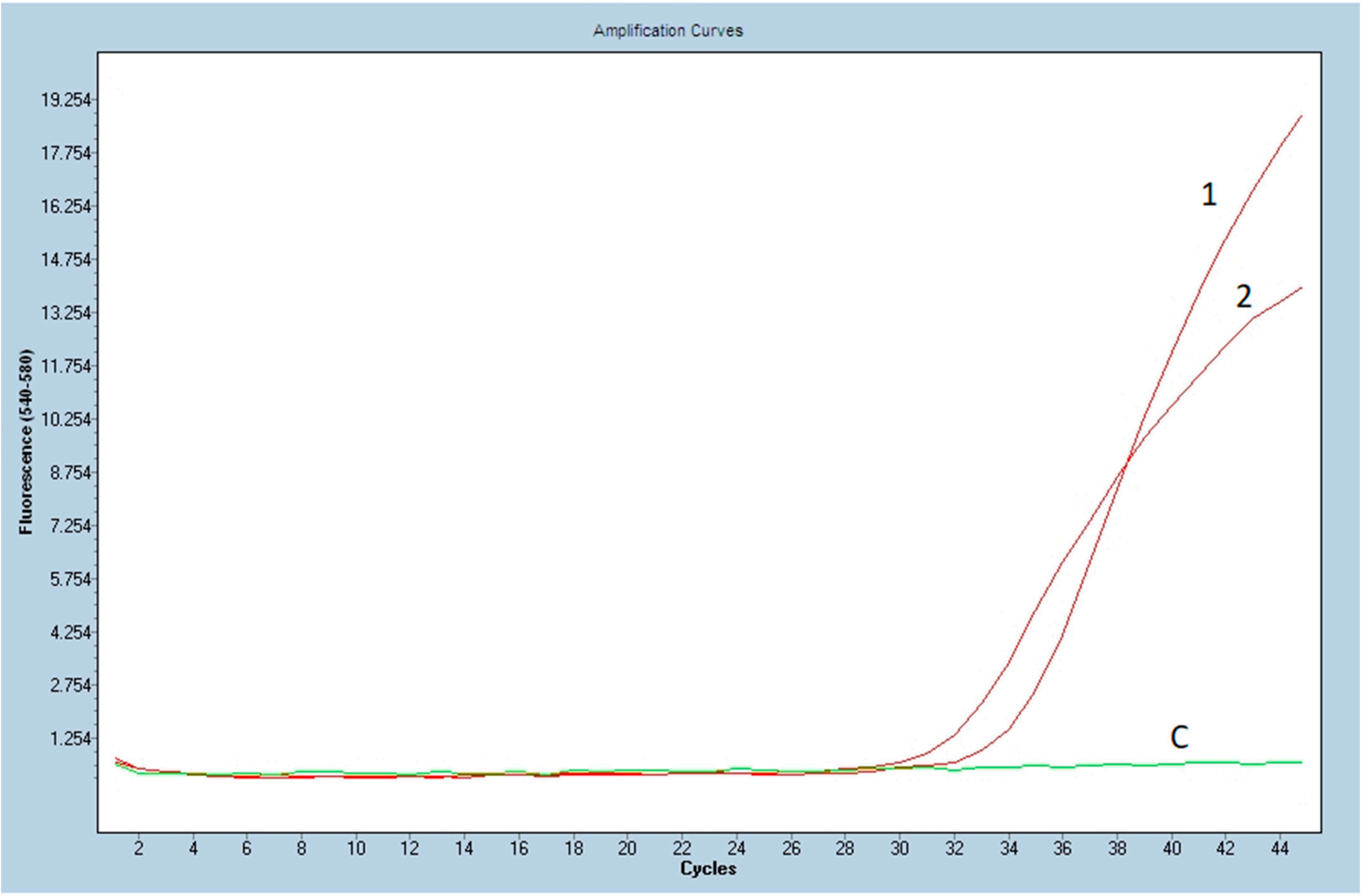

(b)

1, 2—samples numbers, C—calibrator
